# Mutation-Associated Phenotypic Heterogeneity in Novel and Canonical PIK3CA Helical and Kinase Domain Mutants

**DOI:** 10.3390/cells9051116

**Published:** 2020-04-30

**Authors:** Arman Ali Ghodsinia, J-Ann Marie T. Lego, Reynaldo L. Garcia

**Affiliations:** Disease Molecular Biology and Epigenetics Laboratory, National Institute of Molecular Biology and Biotechnology, University of the Philippines Diliman, Quezon City 1101, Philippines; arman.ghodsinia@oncology.ox.ac.uk (A.A.G.); jtlego@up.edu.ph (J-A.M.T.L.)

**Keywords:** PIK3CA, colorectal cancer, EGFR pathway, tumor heterogeneity

## Abstract

Phosphatidylinositol 3-kinase, catalytic subunit alpha (PIK3CA) is an oncogene often mutated in colorectal cancer (CRC). The contribution of PIK3CA mutations in acquired resistance to anti-epidermal growth factor receptor (EGFR) therapy is well documented, but their prognostic and predictive value remain unclear. Domain- and exon-specific mutations are implicated in either favorable or poor prognoses, but there is paucity in the number of mutations characterized outside of the mutational hotspots. Here, two novel non-hotspot mutants—Q661K in exon 13 and C901R in exon 19—were characterized alongside the canonical exon 9 E545K and exon 20 H1047R mutants in NIH3T3 and HCT116 cells. Q661K and E545K both map to the helical domain, whereas C901R and H1047R map to the kinase domain. Results showed variable effects of Q661K and C901R on morphology, cellular proliferation, apoptosis resistance, and cytoskeletal reorganization, with both not having any effect on cellular migration. In comparison, E545K markedly promoted proliferation, survival, cytoskeletal reorganization, migration, and spheroid formation, whereas H1047R only enhanced the first three. In silico docking suggested these mutations negatively affect binding of the p85 alpha regulatory subunit to PIK3CA, thereby relieving PIK3CA inhibition. Altogether, these findings support intra-domain and mutation-specific variability in oncogenic readouts, with implications in degree of aggressiveness.

## 1. Introduction

Phosphatidylinositol 3-kinase, catalytic subunit alpha (PIK3CA) is an oncogene mutated in 10% to 25% of colorectal cancers (CRCs) [1,2,3,4]. Gain-of-function mutations in PIK3CA allow it to activate several signaling pathways, most notably the PIK3CA– Protein Kinase B alpha (AKT) pathway downstream of the epidermal growth factor receptor (EGFR), to promote tumorigenesis. Hotspot mutations in PIK3CA lie in exon 9 (E542K and E545K) in the helical domain and exon 20 (H1047R) in the catalytic kinase domain. These mutations may co-exist with Kirsten Rat Sarcoma Viral Oncogene Homolog (KRAS) and B-Raf Proto-Oncogene, Serine/Threonine Kinase (BRAF) mutations to exert different oncogenic effects and responses to anti-EGFR therapy [5]. Due to change in protein structure induced by the mutations, exon 9 mutations rely on RAS- Guanosine Triphosphate (RAS-GTP) binding to induce transformation, whereas exon 20 mutations are independent of RAS-GTP binding and rely on p85 binding instead [6]. Studies have also correlated exon 20 mutations but not exon 9 mutations to poor response to anti-EGFR therapy in metastatic colorectal cancer [2,7,8]. In uterine endometrial adenocarcinomas, PIK3CA mutations in the kinase domain (exon 20) are associated with adverse prognostic parameters [9].

Various studies suggest that the phenotypic outcome of helical and kinase domain mutations are cell type- and mutation-specific [10,11,12]. Pang et al. [10] reported that the E545K helical domain mutation made cells more significantly chemotactic in the MDA-MB-231 Human Caucasian breast adenocarcinoma cell line, as well as increasing rate of intravasation and extravasation in vivo compared to the H1047R kinase domain mutation. In normal human urothelial cells, the E545K mutation enhanced cellular proliferation and survival more than H1047R [11]. Meyer et al. [12] observed that H1047R was more efficient in forming mice mammary tumors compared to E545K. Indeed, there is a wealth of information regarding exon 9 and 20 mutations and most sequencing studies point to these mutations as occurring most frequently in the population. However, the majority of these studies have been performed in developed nations, and hence the mutational spectrum identified has most likely been biased towards the populations under study [13,14,15,16]. Consequently, non-hotspot mutations have not been characterized as much [17]. Studies that have functionally characterized non-hotspot mutations have mostly been limited to the assessment of colony forming ability, lipid kinase activity, and AKT phosphorylation [16,18,19,20,21]. Given the myriad of signaling pathways affected by PIK3CA, which include both AKT-dependent and -independent mechanisms [22,23,24,25,26], there is a need to phenotypically characterize the oncogenic readouts of non-hotspot mutations in more cancer hallmarks. These mutations may engage different signaling pathways, confer different cancer phenotypes, and may or may not be associated with resistance to anti-EGFR therapy.

This study reports the phenotypic characterization of two novel non-hotspot PIK3CA mutations, namely, Q661K (NM_006218.1: c.1981C > A) located in exon 13 and C901R (Pfam entry PF00454) located in exon 19 [26,27], alongside the canonical mutants E545K (exon 9) and H1047R (exon 20). Q661K has been reported in the Catalogue of Somatic Mutations in Cancer (COSMIC) database [28]. C901R is found in the Human Cancer Proteome Variation [29] and the Epithelial-to-Mesenchymal Gene [30] databases, and is also a distinct and separate entry from the mutants C901F and C901Y [29]. Both Q661K and C901R remain uncharacterized. Notably, Q661K was also identified from an ongoing targeted next generation sequencing study of young-onset sporadic colorectal cancer tissue samples obtained from the Philippine General Hospital, University of the Philippines, Manila [31]. Similar to E545K, Q661K maps to the helical domain of PIK3CA. C901R and H1047R both map to the kinase domain. The mutations were characterized and compared for their effects on cellular proliferation, survival, migration, spheroid formation, general morphology, and cytoskeletal organization.

## 2. Materials and Methods

### 2.1. Cloning and Site-Directed Mutagenesis of Wild Type and PIK3CA Mutants

Wild type (WT) PIK3CA (NM_006218.1) was amplified from cDNA template generated from human kidney 2 cells (HK2; cat. no. CRL-2190, American Type Culture Collection (ATCC), Manassas, Virginia, USA) RNA. The forward (F) and reverse (R) primers used to amplify the PIK3CA constructs are listed in Table 1.

Each PCR mixture contained a final concentration of 1X PCR buffer (Titanium Taq PCR buffer, Clontech Laboratories, Inc., Mountain View, CA, USA), 0.125 mM of each deoxynucleoside triphosphate (iNtRON Biotechnology, Sangdaewon-Dong, Jungwon-Seongnam, Korea), 2 mM each of the appropriate forward and reverse primers, 1X Taq polymerase (Titanium Taq polymerase, Clontech Laboratories), and 50 ng of the appropriate template (cDNA for WT PIK3CA, and cloned pTargeT-PIK3CA WT for the mutations). All PCR reactions were carried out using the C1000 Touch Thermal Cycler (Bio-Rad Laboratories, Inc., Hercules, CA, USA). The PCR mixtures were initially denatured at 95 °C for 5 min. Afterwards, they were subjected to 25 cycles of denaturation at 95 °C for 30 s, annealing at 55 °C for 30 s, and extension at 72 °C for 1.5 min. 

For each mutant construct, fragments from the first round PCR were fused via splicing-by-overlap extension PCR. Briefly, 25 ng of each fragment was used as template with WT-PIK3CA-F and WT-PIK3CA-R as primers, using similar PCR conditions as described above, except for two changes: the extension time was adjusted to 3 min, and an additional final extension step at 72 °C for 10 min was added. The final extension step was added to ensure the addition of A-overhangs for TA-ligation into the pTargeT mammalian expression vector (Promega Corporation, Madison, Wisconsin, USA). All constructs were verified as error-free via Sanger sequencing. 

### 2.2. Cell Culture and Transfection

NIH3T3 mouse fibroblast cells (cat. no. CRL-1658; ATCC) were cultured in Dulbecco’s modified Eagle’s medium (DMEM; Gibco; Thermo Fisher Scientific, Inc., Waltham, MA, USA) supplemented with 10% newborn calf serum (NBCS; Gibco; Thermo Fisher Scientific, Inc.). HCT116 human colon cancer cells (cat. no. CCL-247; ATCC) were cultured in Roswell Park Memorial Institute 1640 medium (RPMI-1640; Gibco; Thermo Fisher Scientific, Inc.) supplemented with 10% fetal bovine serum (FBS), 50 U/mL penicillin/streptomycin, and 2.0 g/L sodium bicarbonate. Both cell lines were incubated in a 37 °C humidified chamber with 5% CO_2_. 

NIH3T3 cells were transfected using the 10 µL Neon Transfection System (Invitrogen, Thermo Fisher Scientific Inc.) according to the manufacturer’s instructions. Briefly, 900,000 cells were mixed with 2 µg of plasmid and electroporated at a pulse voltage of 1400 volts, pulse width of 20 milliseconds, and pulse number of 2. The cells were then seeded in a 12-well plate containing DMEM supplemented with 10% NBCS and left to adhere overnight in a 37 °C humidified chamber with 5% CO_2_. Transfection parameters were optimized to achieve 80–90% efficiency for all experiments.

HCT116 cells were transfected using FugeneHD (Promega) according to the manufacturer’s instructions. Transfection efficiencies of 70–80% for all experiments were consistently obtained using the following plasmid to transfection reagent ratio: 2000 ng DNA/5 μL FugeneHD in 24-well plates, and 200 ng DNA/0.5 μL FugeneHD in 96-well and 48-well plates.

Proof of expression of the constructs is included in Figure S1.

### 2.3. Morphological Characterization

Forty-eight hours post-transfection, transfected NIH3T3 cells were photographed at 100× magnification using an Olympus IX71 inverted fluorescence microscope (Olympus Corporation, Tokyo, Japan). For each transfected well, at least four random fields containing a minimum of 100 cells were imaged. Cells with prominent filopodial extensions and cells that were reduced in size, round, and refringent were counted using ImageJ software [32]. The total number of cells showing prominent cellular protrusions and cells exhibiting roundedness and birefringence were divided by the total cell count determined via the cell counter plugin of ImageJ software [32]. 

To measure the length of cellular protrusions, the Simple Neurite Tracer plugin of Fiji software [33] was used. Briefly, each protrusion was traced from the center of the nucleus to the tip of the extension. The total length of all cellular protrusions in each field was divided by the corresponding field’s total cell count determined via the cell counter plugin of ImageJ software [32], and the resulting mean lengths were compared to wild type and vector-only control.

### 2.4. Actin Cytoskeletal Staining and Analysis

Twenty-four hours post-transfection, transfected NIH3T3 cells were trypsinized from the 12-well plate and seeded in an 8-well glass chamber slide (EMD Millipore, Burlington, MA, USA) at a density of 8000 cells per well. Forty-eight hours post-transfection, the cells were fixed with 4% paraformaldehyde for 20 min. They were then permeabilized with 0.1% Triton X-100 in 1× Phosphate Buffered Saline (PBS) for 20 min, and blocked with 1% bovine serum albumin (Sigma-Aldrich; Merck KGaA, Darmstadt, Germany) in 1× PBS for 20 min. The cells were then stained with 100 µL of 0.165 µM of Alexa Flour 488 Phalloidin (Thermo Fisher Scientific, Inc.) in 1× PBS for 30 min, and counterstained with 100 µL of 1 µg/mL Hoechst 33342 for 15 min. Stained cells were mounted in 70% glycerol in 1× PBS. All steps were performed at room temperature, and the cells were washed thrice with 1× PBS in between steps.

Slides were observed under a fluorescence microscope (Olympus IX83; Olympus Corporation, Tokyo, Japan) at 400× magnification, using the green fluorescent filter (λex/λem: 490/525 nm) to visualize stained filamentous actin structures, and the blue fluorescent filter (λex/λem: 355/465 nm) to visualize the nuclei.

### 2.5. Scratch Wound Healing Assay

NIH3T3 cells were transfected in a 12-well plate. Twenty-four hours post-transfection, cells were reseeded in a 96-well half area plate at a density of 15,000 cells per well in triplicate. Upon reaching full confluence, a scratch was made in each well using a sterile pipette tip. The cells were then maintained in DMEM supplemented with 2.5% NBCS. Migration of cells into the wound area was photographed every hour for 21 h via a motorized time-lapse microscope (Olympus IX83; Olympus Corporation, Tokyo, Japan).

HCT116 cells were seeded at a density of 10,000 cells per well in a 96-well plate and then transfected 36 h later. Upon reaching full confluence, a scratch was made in each well using a sterile pipette tip. The cells were then maintained in RPMI-1640 supplemented with 2% FBS. Migration of cells into the wound area was photographed at 0 h and 24 h post-scratch using Olympus IX71 inverted fluorescence microscope (Olympus Corporation, Tokyo, Japan).

The gap area for each time point was determined using ImageJ software [32]. The migration rate (µm^2^/h) for each setup was determined by obtaining the slope of the trend line generated after the gap area (µm^2^) was plotted against time (h).

### 2.6. Cell Proliferation Assay

NIH3T3 cells were transfected in a 12-well plate. Twenty-four hours post-transfection, cells were reseeded in 96-well plates at a density of 2500 cells per well in triplicate. The cells were then maintained in DMEM supplemented with 2.5% NBCS.

HCT116 cells were seeded at a density of 2500 cells per well in 48-well plates and transfected 36 h later. Twenty-four hours post-transfection, cells were maintained in RPMI-1640 supplemented with 0.5% FBS.

The number of metabolically active cells per setup was determined at 48 and 72 h post-transfection for NIH3T3 and at 48, 72, and 96 h post-transfection for HCT116, by incubating each well with 10 µL of CellTiter 96 Aqueous One Solution Cell Proliferation Assay reagent (Promega Corporation) until color development. Absorbance values at 490 nm of each setup were measured with a colorimetric plate reader (FLUOstar Omega microplate reader; BMG Labtech, Cary, NC, USA). Cell counts were calculated from a standard curve (number of cells vs. A490) generated using serial dilutions of an untransfected cell suspension. Mean cell counts were calculated per setup for each time-point.

### 2.7. Apoptosis (Caspase 3/7) Assay

NIH3T3 cells were transfected in a 12-well plate. Twenty-four hours post-transfection, cells were reseeded in 96-well plates at a density of 2,500 cells per well in triplicates. Cells were then maintained in DMEM supplemented with 2.5% NBCS. 

HCT116 cells were seeded at a density of 2,500 cells per well in a 96-well plate and transfected 36 h later. Twenty-four hours post-transfection, cells were then maintained in RPMI-1640 supplemented with 0.5% FBS.

The level of caspase 3/7 activity for each setup was determined at 48 h post-transfection for NIH3T3 and at 96 h post-transfection for HCT116 by incubating the cells with 10 µL of Caspase-Glo 3/7 assay reagent (Promega Corporation). The plates were incubated at room temperature for 3 h and then the luminescence per well was measured using a plate reader (FLUOstar Omega microplate reader; BMG Labtech). Luminescence readings were normalized to the number of viable cells determined via a cell proliferation assay performed concurrently.

### 2.8. Western Blot Analyses

NIH3T3 and HCT116 cells were transfected in 12-well plates. Transfected NIH3T3 cells were cultured in DMEM supplemented with 2.5% NBCS at 24 h post-transfection. At 60 h post-transfection, transfected NIH3T3 cells were further serum starved by culturing them in DMEM supplemented with 1% NBCS. Transfected HCT116 cells were cultured in RPMI-1640 supplemented with 10% FBS throughout.

At 72 h post-transfection, proteins were harvested for SDS-PAGE and Western blotting. Cells were lysed with radioimmunoprecipitation lysis buffer (150 mM NaCl, 0.5% sodium deoxycholate, 0.1% sodium dodecyl sulphate, 50 mM Tris (pH 8.0)) supplemented with Halt protease and phosphatase inhibitor cocktail (Thermo Fisher Scientific Inc.). For gel electrophoresis, 20 µg of protein were run on a 7.5% SDS-PAGE gel at 100 V for 1 h. Proteins were blotted for 24 h at a constant current of 40 mA. Membranes were blocked with 5% w/v non-fat dry milk in Tris-buffered saline with 0.1% v/v Tween-20 (TBST) for 1 h at room temperature; probed overnight with primary antibodies obtained from Cell Signaling Technology (Danvers, MA, USA): anti-PIK3CA (1:1000, cat. no. 4249S), anti-N-cadherin (1:1000, cat. no. 14215S), anti-E-cadherin (1:1000, cat. no. 3195S), and anti- Glyceraldehyde 3-phosphate dehydrogenase (GAPDH; 1:1000, cat. no. 2118S); washed with TBST; and incubated with the appropriate secondary antibodies obtained from Thermo Fisher Scientific Inc.: goat anti-mouse Immunoglobulin G (IgG, H + L; 1:3000, cat. no. 31430) and goat anti-rabbit IgG (1:3000, cat. no. 31460) for 1 h at room temperature. Signals were developed with enhanced chemiluminescence substrate and imaged using the ChemiDoc Touch Imaging System (Bio-Rad Laboratories, Inc., Hercules, CA, USA). Densitometric analysis of digitized band intensities was performed using GelQuant.NET software (v1.8.2) provided by biochemlabsolutions.com. Gene expression levels were normalized against GAPDH expression.

### 2.9. Spheroid Formation Assay

Twenty-four hours post-transfection, transfected NIH3T3 cells were trypsinized from the 12-well plate and seeded in a 96-well round bottom ultra-low-attachment spheroid plate (Corning Incorporated, New York City, NY, USA) at a density of 2,500 cells per well in triplicate. The cells were maintained in DMEM supplemented with 2.5% NBCS. The spheroids were photographed at 40× magnification using an Olympus IX71 inverted fluorescence microscope (Olympus Corp., Tokyo, Japan) at 96 h post-transfection. The diameter of the spheroids was then measured using ImageJ software [32].

### 2.10. Bioinformatics-Based Prediction of the Functional Impact of PIK3CA Mutations

The functional impact of the PIK3CA Q661K and C901R mutations was predicted through four sequence-based algorithms: Polymorphism Phenotyping 2 (Polyphen-2; version 2) (accessed on 20 August 2019; http://genetics.bwh.harvard.edu/pph2/) [34], Mutation Assessor (release 3) (accessed on 20 August 2019; http://mutationassessor.org/r3/) [35], Functional Analysis Through Hidden Markov Models (FATHMM; version 2.3) (accessed on 20 August 2019; http://fathmm.biocompute.org.uk/) [36], and Sorting Tolerant From Intolerant (SIFT; version 5.2.2) (accessed on 20 August 2019; https://sift.bii.a-star.edu.sg/www/SIFT_seq_submit2.html) [37].

To generate homology models of the H1047R, E545K, Q661K, and C901R mutations, the Swiss-model [38] webserver (accessed on 20 August 2019; https://swissmodel.expasy.org/interactive) was utilized. The template used for homology modelling was the crystal structure of native PIK3CA in complex with niSH2 (N-terminal Src Homology 2 / inter Src Homology 2) domain of p85 alpha (Protein Data Bank or PDB ID: 4L1B) [39]. Using BIOVIA Discovery Studio Visualizer (Dassault Systèmes BIOVIA, San Diego, CA, USA), the models of the mutations were superimposed to the wild type and the global main chain root mean square distance (RMSD), and overlay similarity (OS) were calculated.

The impact of the mutations on the ability of the niSH2 domain of p85 alpha to form a dimer with PIK3CA was assessed using the pyDOCK webserver (accessed 22 August 2019; https://life.bsc.es/pid/pydockweb) [40,41]. The pyDock scores of the best poses were compared across all mutations. The interactions between the interface residues of the PIK3CA–p85 alpha dimer were analyzed with PDBePISA (version 1.52) (accessed on 25 August 2019; https://www.ebi.ac.uk/pdbe/pisa/) [42].

### 2.11. Statistical Analyses

All data were analyzed using unpaired one-tailed *t*-test to compare differences between the vector only control/wild type and each of the mutants. Data from quantitative experiments were represented as mean ± standard deviation. Significant values were represented as * *p* < 0.05, **p < 0.01, and *** *p* < 0.001.

## 3. Results

### 3.1. The PIK3CA Mutations Had Variable Effects on Proliferative Rates of NIH3T3 and HCT116 Cells

**Figure 1:**
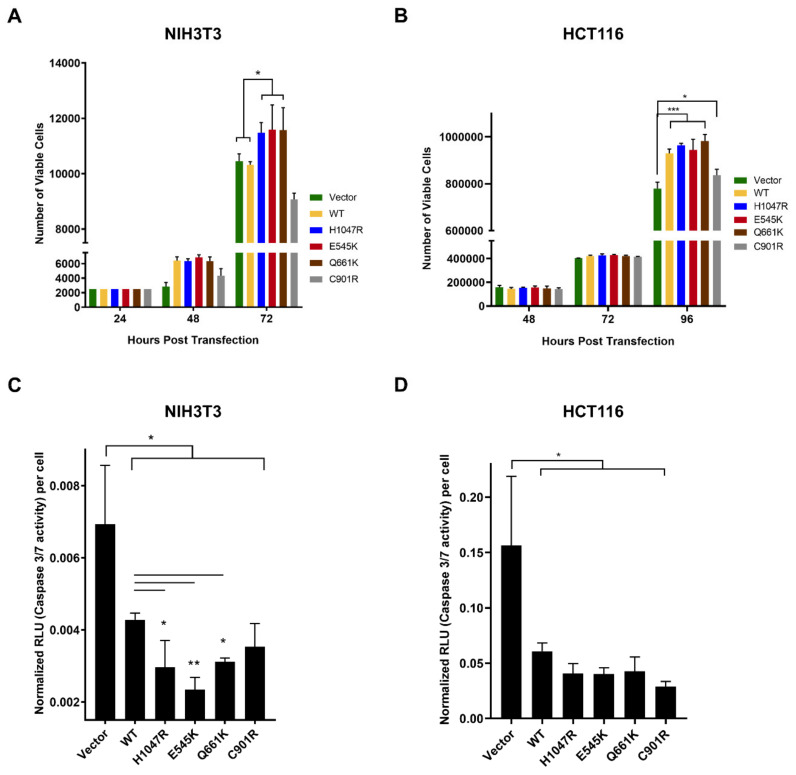
Variable effects of wild type (WT), canonical, and novel PIK3CA mutants on proliferative capacity and apoptosis resistance in NIH3T3 and HCT116 cells. Proliferation rates of (**A**) NIH3T3 and (**B**) HCT116 cells, and caspase 3/7 activity in (**C**) NIH3T3 and (**D**) HCT116 cells transfected with empty vector, wild type PIK3CA, or PIK3CA mutants. Data presented are representative of three independent trials in triplicates and expressed as mean ± standard deviation. * *p* < 0.05, ** *p* < 0.01and *** *p* < 0.001. WT: wild type.

To determine if expression of the PIK3CA mutants can promote cellular proliferation, the number of viable cells per setup was determined at 24, 48, and 72 h post-transfection for NIH3T3 cells and at 48, 72, and 96 h for HCT116 cells. The results in HCT116 were generally consistent with those obtained in NIH3T3 cells (Figure 1A,B). The canonical mutants E545K and H1047R as well as the novel mutant Q661K enhanced proliferative capacity. C901R enhanced proliferation only in HCT116. The effect of the wild type construct in the two cellular backgrounds, however, showed a marked difference. In NIH3T3 cells, WT had no apparent effect on proliferation and was indistinguishable from that of the vector-only control. In HCT116 cells, WT overexpression was able to enhance proliferative capacity. There are at least two plausible explanations for this. HCT116 harbors an endogenous KRAS G13D mutation and it is highly likely that it is able to hyperactivate wild type PIK3CA, which is downstream of KRAS in the signaling pathway; hence, the observed enhanced proliferation. Alternatively, the presence of the endogenous PIK3CA H1047R (in addition to KRAS G13D) and the overexpression of wild type PIK3CA may have a synergistic effect that could have led to enhanced proliferation.

### 3.2. Variable Effects of the Canonical Mutants E545K and H1047R, and the Novel Mutants Q661K and C901R on Apoptosis Resistance in NIH3T3 and HCT116 Cells

PIK3CA is known to promote cell survival [43,44]. To test the capacity of the PIK3CA mutants to inhibit apoptosis, the activity of caspase 3/7 was assessed in transfected cells using the caspase-Glo 3/7 assay. In NIH3T3 cells, overexpression of the Q661K novel mutant and the H1047R and E545K canonical mutants led to a significant reduction in caspase 3/7 activity, indicating resistance to apoptosis (Figure 1C). Among all mutants, E545K had the lowest level of caspase 3/7 activity. Cells overexpressing wild type PIK3CA and the novel C901R mutant showed the highest level of caspase 3/7 activity but still demonstrated resistance to apoptosis compared to vector-only control. In HCT116 cells, the wild type and all mutant constructs also induced resistance to apoptosis, although the degree of inhibition did not vary widely among the different setups (Figure 1D). The NIH3T3 cell line is usually preferred in characterizing oncogenes and their mutant variants because they do not require cooperative complementary mutations to express a transformed phenotype [45]. In addition to the non-cancerous background, this may explain the more resolved differences in degree of resistance to apoptosis among the wild type and mutant setups in NIH3T3 compared with HCT116. 

### 3.3. Novel and Canonical PIK3CA Mutants Induced Gross Morphological Alterations and Enhanced Formation of Pseudopodial Extensions

Gross morphological alterations can be indicative of oncogenic transformation. Transformed NIH3T3 cells typically show decreased size, refringency, pronounced pseudopods, and increased cellular protrusions [46,47]. To determine if the canonical and novel PIK3CA mutations can induce morphological alterations, transfected cells were observed under a brightfield microscope. The percentage of cells showing pronounced extensions, the average length of these cellular extensions, and the percentage of cells exhibiting roundedness and birefringence were also quantified.

**Figure 2:**
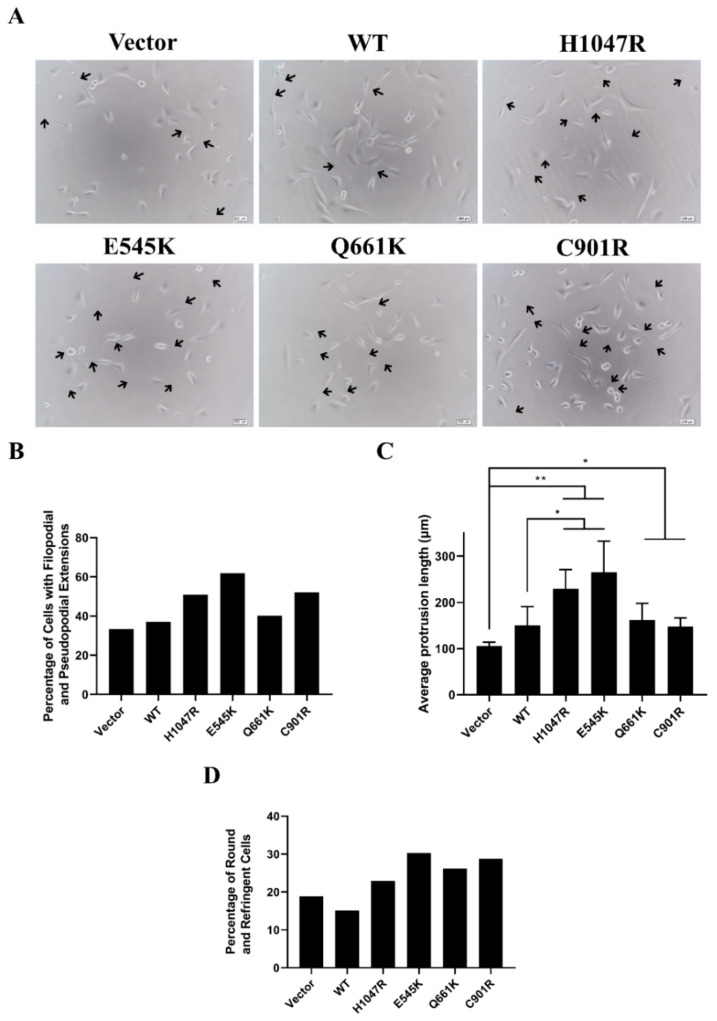
Altered gross morphology of NIH3T3 cells overexpressing PIK3CA mutants. (**A**) Prominent cellular protrusions (black arrows) were observed in cells overexpressing PIK3CA mutants. Scale bar: 200 µm. (**B**) NIH3T3 cells with prominent cellular protrusions were counted and divided by total number of fibroblasts. (**C**) The lengths of cellular protrusions were measured from the center of the nucleus to the tip of the extension. The total length was then divided by the total number of fibroblasts. (**D**) Cells showing decreased size, roundedness, and birefringence were quantified and divided by the total cell count. The percentage of birefringent cells and cells with cellular protrusions, as well as the average length of these protrusions, increased in cells overexpressing the PIK3CA mutations. Data presented are representative of three independent trials in triplicate, and expressed as mean ± standard deviation. * *p* < 0.05 and ** *p* < 0.01. WT: wild type. PIK3CA: phosphatidylinositol 3-kinase, catalytic subunit alpha.

Results indicate that overexpression of the PIK3CA mutants caused decreased size and rounding of cells, refringency, and formation of cellular protrusions (Figure 2). In cells overexpressing the E545K and H1047R hotspot mutants, there was a dramatic increase in the percentage of cells showing pronounced cellular protrusions compared to the rest of the setups. These cells also had the longest average length of cellular protrusions. Overexpression of C901R enhanced formation of cellular protrusions to levels comparable with that of H1047R and showed an average protrusion length similar to Q661K and wild type setups. Q661K had marginal increase in the number of protrusions and average length compared to the wild type and vector only control. Consistent with these findings, overexpression of all four mutants also increased the percentage of round and refringent cells compared to the vector-only control.

### 3.4. Cells Overexpressing Wild Type PIK3CA as well as Novel and Canonical PIK3CA Mutants Were Highly Depolarized with Long Cellular Protrusions

**Figure 3:**
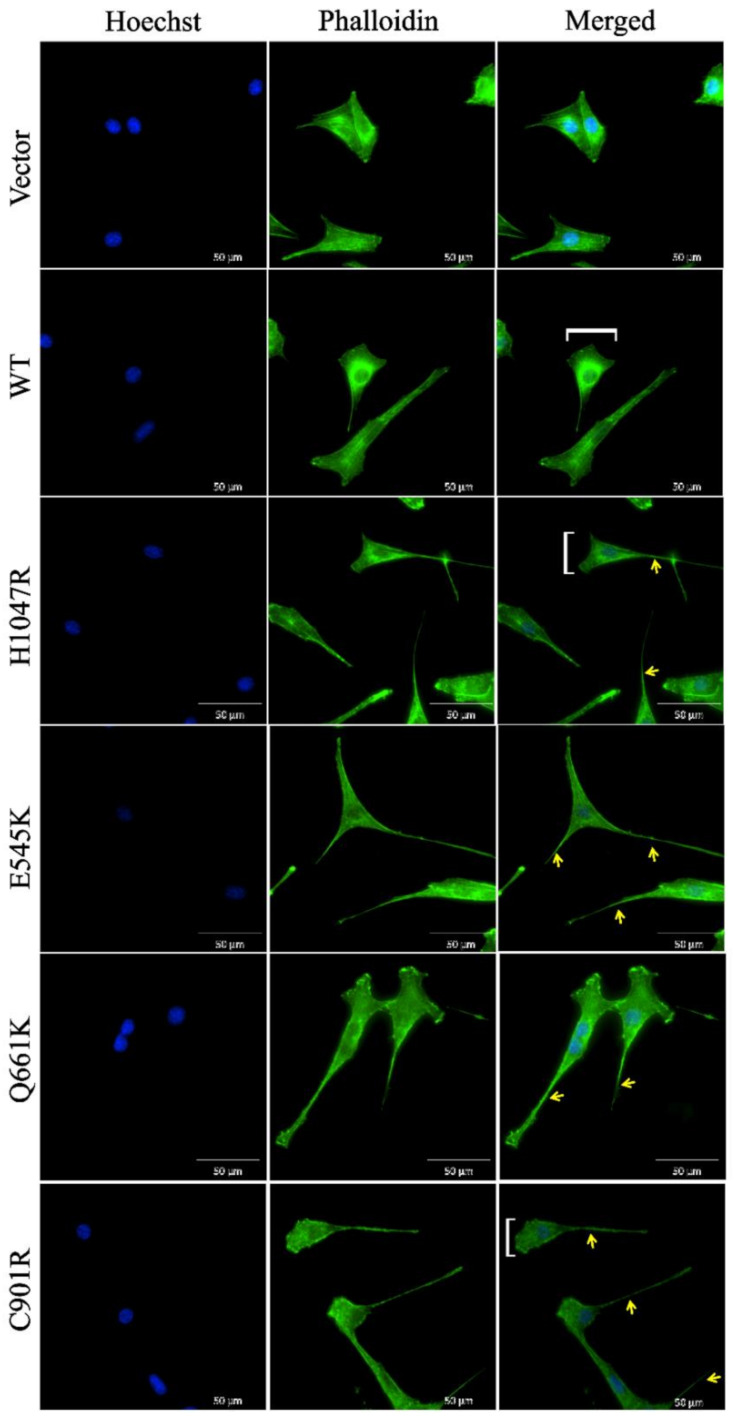
Cytoskeletal reorganization of cells overexpressing wild type, novel, and canonical PIK3CA mutations. White brackets show prominent migrating front. Yellow arrows point to polymerized actin of pseudopod extensions. Scale bar: 50 µm. Data presented are representative of three independent trials in triplicates. WT: wild type.

To determine if wild type PIK3CA and the novel and canonical PIK3CA mutants can induce cytoskeletal reorganization, F-actin in transfected cells were visualized via phalloidin staining and fluorescence microscopy. In both wild type and mutant setups, the cells were dramatically depolarized, with prominent leading edge, lamellipodia, and long cellular protrusions (Figure 3). All these cytoplasmic changes suggest a highly motile phenotype propelled by dynamic actin networks.

To evaluate whether the morphological changes induced by the PIK3CA mutants are due, at least in part, to epithelial-to-mesenchymal transition (EMT), the expression levels of E-cadherin and N-cadherin were examined in transfected HCT116 cells, an epithelial cancer cell line. Despite its cancerous cellular background, it is still possible to augment and observe EMT changes in HCT116 after transfection with oncogenic mutants or upregulation of cellular oncogenes [48,49]. Western blot results showed consistent partial EMT only for the E545K mutant, that is, a consistent apparent decrease in E-cadherin, but inconclusive results for N-cadherin. The wild type and other mutant constructs showed inconsistent results across trials, which may suggest no effect on EMT markers (Figure S2). This is not totally surprising given that many aspects that contribute to morphological changes (e.g., cytoskeletal reorganization, elaboration of cellular processes and protrusions, E- to N-cadherin switch) are in fact controlled by distinct signaling pathways [50,51,52,53].

### 3.5. E545K, but not the Other PIK3CA Mutants, Enhanced Migration of NIH3T3 Cells

**Figure 4:**
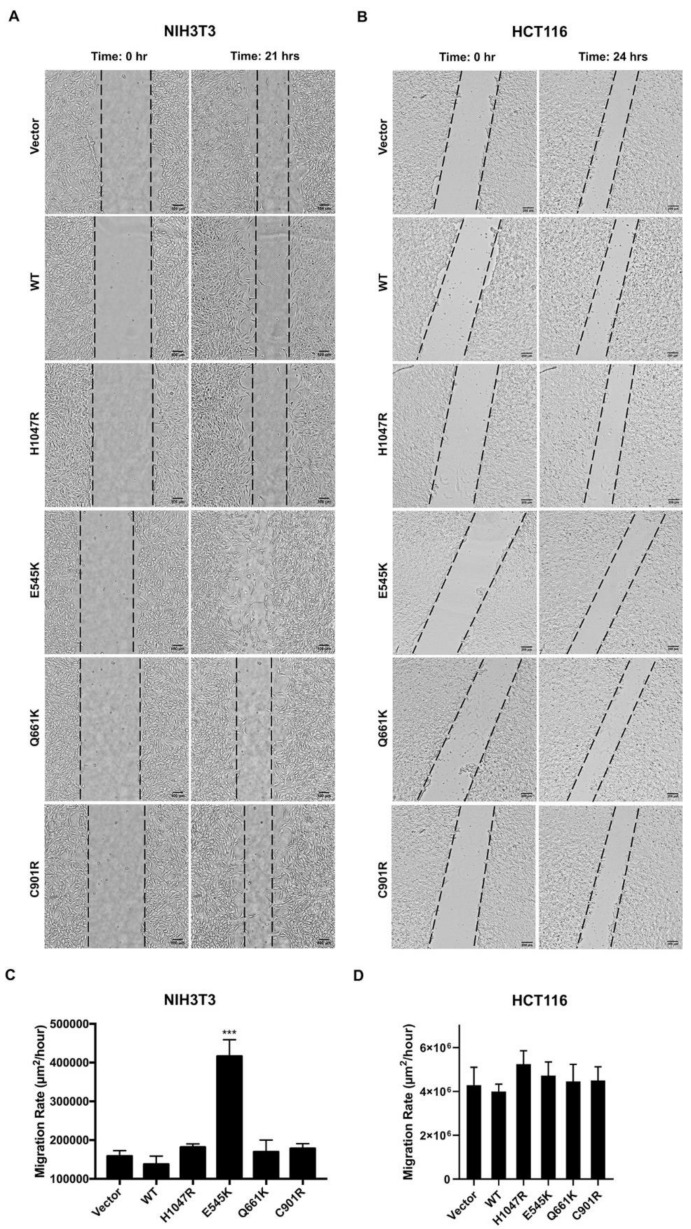
Effect of wild type and PIK3CA mutations on cellular migration in vitro. Confluent monolayers of (**A**) NIH3T3 and (**B**) HCT116 cells were scratched and brightfield images of wound closure were taken for at least a 21 h period post-scratch. Compared with the vector and wild type controls, narrower scratch gaps were noticeable only for NIH3T3 cells overexpressing the PIK3CA E545K hotspot mutant. No significant change was observed for the rest of the PIK3CA mutants in NIH3T3 and HCT116 cells. Scale bars: 100 μm (NIH3T3) and 200 μm (HCT116). Migration rates of (**C**) NIH3T3 and (**D**) HCT116 cells were measured by obtaining the slope of the trend line generated when the wound area was plotted against time for every time point (µm^2^/h). Data presented are representative of three independent trials in triplicate, and expressed as mean ± standard deviation. *** *p* < 0.001. WT: wild type; PIK3CA: phosphatidylinositol 3-kinase, catalytic subunit alpha.

Given that overexpression of PIK3CA mutations was able to alter cellular morphology and cytoskeletal actin organization, effects of the mutations on cellular migration were also determined. Confluent monolayers were scratched with a sterile pipette tip, and migration of cells into the wound area was monitored for 21 (NIH3T3) or 24 h (HCT116). In NIH3T3 cells, only the E545K canonical mutation was able to significantly promote cellular migration compared to wild type and vector-only controls (Figure 4A,C). In HCT116 cells, no marked differences in migratory capacity were observed across setups at 24 h post-scratch (Figure 4B,D). The different results obtained for E545K may have been due to differences in cellular background. As discussed in Section 3.2 above, NIH3T3 cells are better able to express a transformed phenotype, even in the absence of cooperative complementary mutations [45].

The lack of an effect on cellular migration is not inconsistent with the observed cytoskeletal reorganization induced by all the mutants. There are multiple, complex signaling events integrated to bring about migration. Among the many players involved are the Rho GTPases, integrins, phosphoinositides, RAS proteins, and numerous kinases [54]. Although reorganization of the cytoskeleton is an early event necessary for movement, adhesion-dependent migration is a distinct process altogether.

### 3.6. E545K Canonical Mutation Enhanced Spheroid Formation in NIH3T3 Cells

**Figure 5:**
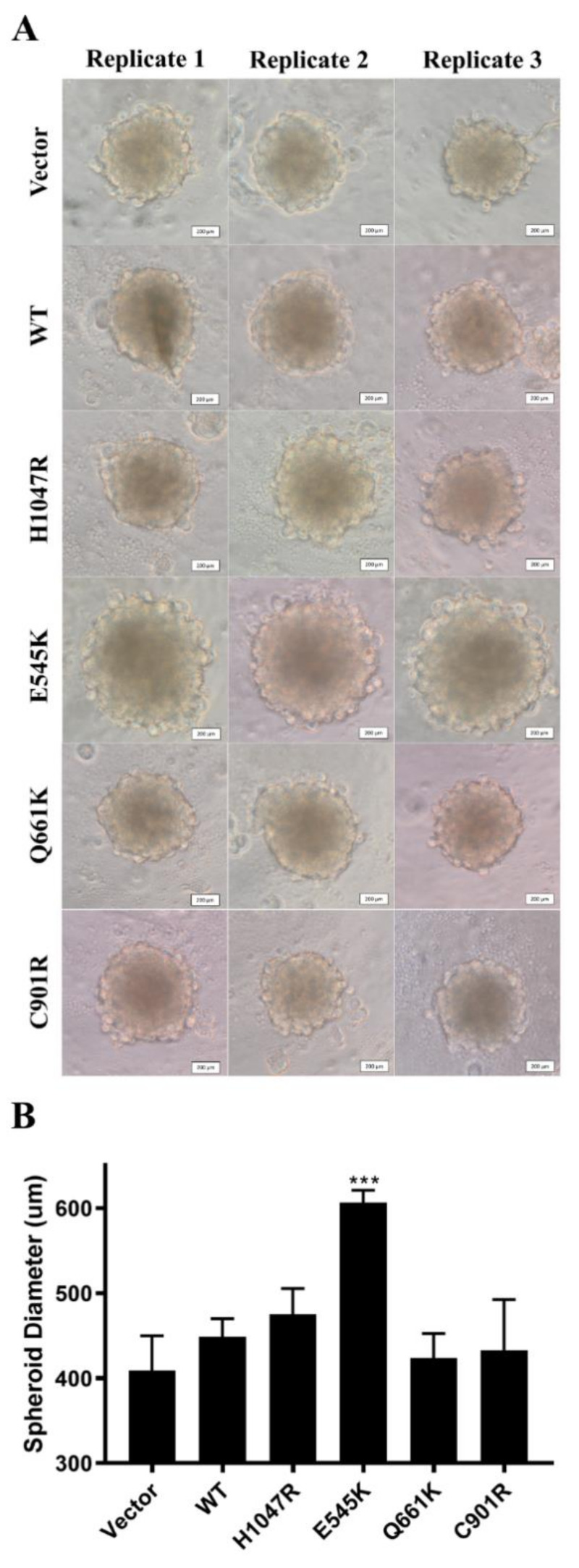
The E545K hotspot mutation, but not the other PIK3CA mutants and wild type, was able to enhance spheroid formation of NIH3T3 cells in vitro. (**A**) Micrographs of the spheroids at 96 h post-transfection show that there was a noticeable increase in the diameter of spheroids overexpressing the E545K hotspot mutation. Scale bar: 200 µm. (**B**) Spheroid diameters were measured at 96 h post-transfection. Data presented are representative of three independent trials in triplicate, and expressed as mean ± standard deviation. *** *p* < 0.001. WT: wild type; PIK3CA: phosphatidylinositol 3-kinase, catalytic subunit alpha.

Tumors rely on cell–cell and cell–extracellular matrix (ECM) interactions for growth and metastasis [55]. Because there is a significant reduction of these interactions in two-dimensional cultures, it is possible that the phenotype of PIK3CA mutant overexpression could be irreproducible in vivo. To determine whether the results obtained thus far could be replicated in a model that mimics the spatial organization of tumor cells, NIH3T3 cells overexpressing the PIK3CA mutations were grown as three-dimensional spheroids. Only the E545K canonical mutant promoted significant spheroid formation at 96 h post-transfection (Figure 5). The H1047R canonical mutant, as well as the novel mutants Q661K and C901R, did not show any significant differences in size when compared to the wild type and vector-only controls.

### 3.7. In Silico Prediction of the Impact of PIK3CA Mutations on Protein Structure and p85 Alpha-niSH2 Binding

To predict if the mutations can impact protein function, four sequence-based platforms were used (Table 2): polyphen-2, which predicts on the basis of physical and comparative approaches [34]; mutation assessor, which utilizes evolutionary conservation of the affected amino acids in protein homologs [35]; FATHMM, which applies a combination of sequence conservation with hidden Markov models [36]; and SIFT, which relies on sequence homology and the physical properties of amino acids [37]. Apart from the FATHMM prediction, the other three platforms predicted Q661K to be benign and have a minimal effect on protein function. Meanwhile, all four platforms predicted C901R to be oncogenic and have a significant effect on protein function.

**Figure 6:**
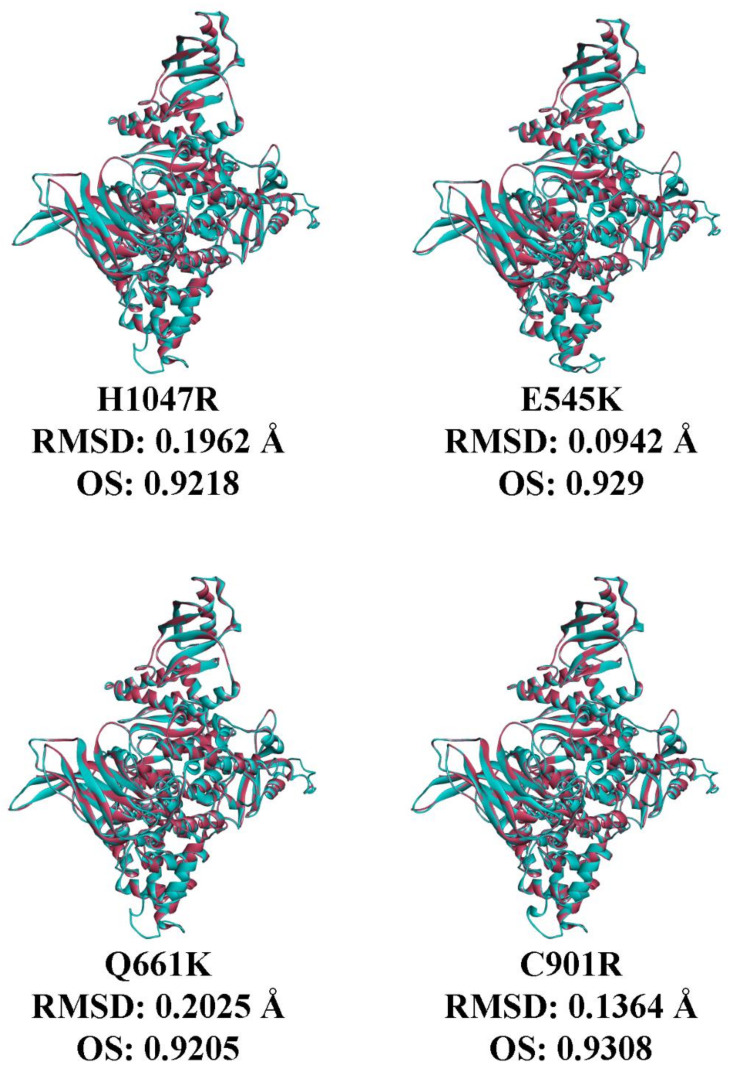
Superposition of modeled PIK3CA mutations (cyan ribbon) with the solved crystal structure of human wild type PIK3CA (PDB ID: 4L1B; red ribbon). Global main chain root mean square distance (RMSD) and overlay similarity (OS) values were also predicted via sequence alignment function for each mutant in comparison with the wild type. RMSD: root-mean-square distance; OS: overlay similarity; PIK3CA: phosphatidylinositol 3-kinase, catalytic subunit alpha.

Protein models of the mutations were generated with Swiss-model webserver. The models were superimposed to the wild type, and the RMSD and OS were calculated (Figure 6). As expected, the RMSD values for all mutations did not exceed 0.25 Å, as single amino acid changes are not expected to significantly alter protein structure. Among all the mutations, the rare Q661K mutation had the highest RMSD and lowest OS compared to wild type. This suggests that the amino acid change at codon 661 led to the most significant deviation in C-alpha carbon backbone, as well as overall protein conformation, possibly even altering the conformation of residues at the PIK3CA–p85 alpha interface. Nonetheless, the rest of the mutations only slightly differed from Q661K in terms of RMSD and OS, suggesting that they also subtly altered protein structure.

**Figure 7:**
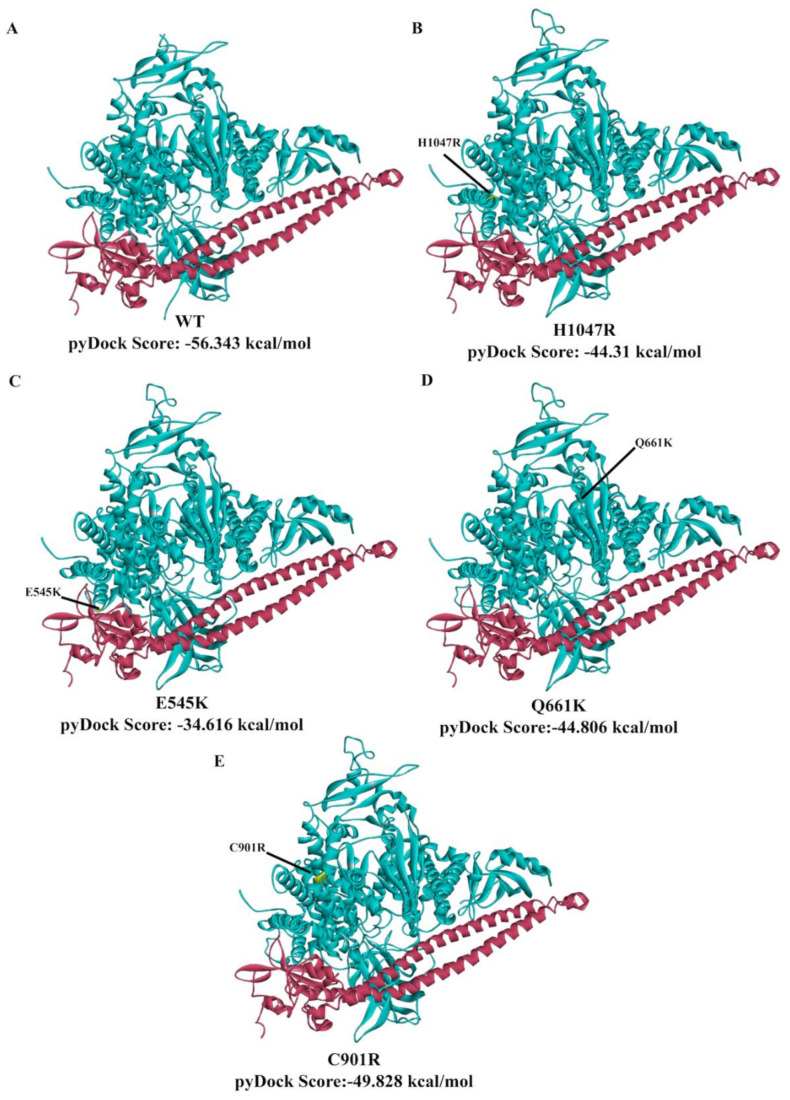
Simulated interactions of niSH2 domain of p85 alpha to wild type and mutant PIK3CA yielded variable binding energies. The solved X-ray crystal structure of human wild type PI3KCA (**A**) PDB 4L1B was used to build mutant homologues of (**B**) H1047R, (**C**) E545K, (**D**) Q661K, and (**E**) C901R. The pose with the most favorable and lowest (i.e., most negative) pyDock score is shown for each interaction. The mutated amino acid is highlighted in yellow and labelled accordingly. WT: wild type; PIK3CA: phosphatidylinositol 3-kinase, catalytic subunit alpha.

Docking simulations using pyDock webserver were performed to assess whether the mutations affected the ability of the niSH2 domain of p85 alpha to bind to PIK3CA. pyDock is a protein–protein docking algorithm [40] that uses electrostatics, desolvation energy, and a limited van der Waals contribution to score rigid-body docking poses. On the basis of the pyDock scores (Figure 7), E545K had the least favorable interaction with the niSH2 domain of p85 alpha. This was followed by H1047R, Q661K, C901R, and wild type. Altogether, the observed trend was E545K (least favorable interaction) < H1047R < Q661K < C901R < WT (most favorable interaction). 

To gain some insight into the possible reasons for the differences in docking scores among the PIK3CA mutations, the interactions between interface residues of the PIK3CA and p85 alpha dimers were examined with PDBePISA (Table 3). The E545K-niSH2 p85 alpha dimer, which had the least favorable docking score, had a decrease in both the number of interface residues and buried interface area compared to the PIK3CA wild type-niSH2 p85 alpha dimer. The E545K-niSH2 p85 alpha dimer also gained two additional hydrogen bonds, but also lost four salt bridges that were present in the wild type-niSH2 p85 alpha dimer. This suggests that the salt bridges formed between the E545K–niSH2–p85 alpha interface had a stronger contribution to the binding affinity of the dimer than the hydrogen bonds. This finding is in accordance with the pyDock scoring algorithm, which gives greater value to electrostatics than van der Waals interactions (e.g., hydrogen bonding) in scoring docking poses [41].

Meanwhile, the H1047R-, Q661K-, and C901R-niSH2 p85 alpha dimers had an increase in both the number of interfacing residues and buried interface area compared to the wild type-niSH2 p85 alpha dimer. Like the E545K-niSH2 p85 alpha dimer, they also had an increase in the number of hydrogen bonds and a decrease in the number of salt bridges between the mutants and the niSH2 domain of p85 alpha.

These results could partially explain the trend observed for the pyDock scores. The E545K dimer had the least favorable pyDock score because in addition to losing four salt bridges in the interface, it also had the least number of interface residues and smallest buried surface area among the PIK3CA variants. On the contrary, the H1047R, Q661K, and C901R dimers also lost a number of salt bridges, but gained in the number of interface residues and buried interface area. Altogether, these results align with the trend E545K (least favorable interaction) < H1047R < Q661K < C901R < WT (most favorable interaction).

## 4. Discussion

Previous studies suggest that the impact of PIK3CA exon 9 and 20 mutations are cell type- and mutation-specific [10,11,12]. Many PIK3CA mutant cancer cell lines have also been shown to exhibit transformed phenotypes despite displaying minimal AKT activation, suggesting AKT-independent mechanisms that must be considered [16,18,19,20,21]. Indeed, the identification of rare non-hotspot PIK3CA mutations in deep sequencing projects worldwide warrants their functional characterization. Due to multiple signaling pathways affected by PIK3CA, rare mutations may transmit their effects through different effectors, such as PDK1, SGK3, and RAC, among others, to exert their oncogenic outputs [22,23,24,25]. Further, the majority of previous sequencing studies have been performed using patient samples from developed nations, and hence the mutational spectrum identified could be biased towards the population under study [14,15,16,17,18]. Ethnic nuances have already been described for KRAS mutants [14,15,57], and it is highly likely that the PIK3CA mutation spectrum may also reveal such differences.

Intratumor heterogeneity, which refers to the coexistence of cell clones harboring variable genetic mutations within a tumor, is another reason for characterizing novel and rare PIK3CA mutations. Intratumor heterogeneity is implicated in failure of some biomarkers to predict tumor response to chemotherapy [58,59]. Because only a small sample from a tumor tissue is used in a biopsy, only the most frequently occurring mutations are detected. Rare mutations present in only a small subset of cells, which could be chemoresistant, remain undetected, and contribute to tumor recurrence after therapy. Further stressing the importance of functionalizing rare mutations is the finding that PIK3CA hotspot mutations differentially impact responses to Mesenchymal Epithelial Transition (MET) receptor tyrosine kinase-targeted therapy [60]. In a similar manner, rare mutations may also differentially impact therapy response.

In this study, two novel non-hotspot mutations, Q661K and C901R, were phenotypically characterized in NIH3T3 and HCT116 cells [28]. PIK3CA mutations in codon 661 were reported to be present in colon cancer, whereas those in codon 901 were reported to be present in colon, breast, and endometrial cancers [61]. Bader et al. [62] reported that in 266 colon cancer samples [26,63], E545 and H1047 mutations occurred at frequencies of 9.8% and 7.1%, respectively, whereas Q661 and C901 mutations both occurred at lower frequencies of 0.4%. Similar trends were observed in breast (580 samples) [26,63,64,65,66], brain (382 samples) [26,67,68], liver (73 samples) [69], stomach (291 samples) [26,65,70], lung (253 samples) [26,69], and ovary (489 samples) [63,65,71] tumors, wherein E545K and H1047R mutations occurred at frequencies ranging from 3.3% to 6.2% for E545 and 1.5% to 14.8% for H1047, whereas both Q661 and C901 mutations were almost undetectable. Comparable trends have been reported in the Cancer Genome Atlas (TCGA) database [72]. Interestingly, Q661K was also reported in a genetic screen of sporadic, young-onset CRC patient samples obtained from the Philippine General Hospital, University of the Philippines, Manila [31].

All four sequence-based prediction platforms predicted C901R to be oncogenic and have a significant effect on PIK3CA function, whereas three out of four predicted Q661K to be benign. Although these platforms do not consider the possible effect of the mutations on protein binding partners, these predictions still prompted us to further characterize the rare mutations in detail. Overexpression of the PIK3CA mutants led to distinct oncogenic phenotypes. In all hallmarks tested, the E545K hotspot mutation was consistently the most aggressive. Meanwhile, the H1047R canonical mutation only exhibited certain cancer hallmarks, namely, resistance to apoptosis, cellular proliferation, and cytoskeletal disorganization. These findings are consistent with previous studies, which suggested distinct oncogenic phenotypes of the E545K and H1047R mutations [10,11,12]. Overexpression of the Q661K and C901R rare mutations also presented distinct cellular phenotypes. The Q661K rare mutation was able to promote cellular proliferation and survival in both NIH3T3 and HCT116 compared to the vector-only and WT controls. The C901R rare mutation, on the other hand, enhanced resistance to apoptosis in NIH3T3 cells compared to vector-only but not WT control, and in HCT116 cells compared to both vector-only and WT controls. The C901R mutant enhanced proliferation only in HCT116 cells relative to vector-only but not WT control. Both Q661K and C901R were able to promote cytoskeletal disorganization.

Although all PIK3CA mutations were able to affect cytoskeletal architecture, only the E545K hotspot mutation was able to enhance cellular migration in wound healing assays, and only in NIH3T3 cells, which are better able to express a transformed phenotype even in the absence of complementary cooperative mutations [45]. Cellular migration is a complex process that involves cytoskeletal reorganization, changes in polarity, formation of cellular extensions, and/or loss of adhesion [54,73]. Hence, the inability of the H1047R hotspot mutation and the Q661K and C901R rare mutations to translate cytoskeletal disorganization to enhanced motility is not unexpected because each mutation may signal through a distinct pathway and may affect different aspects of cellular migration.

Interestingly, these results contradicted the assessment of sequence-based prediction platforms used. Q661K was predicted to be benign and have minimal effect on protein function, and yet was able to show oncogenic properties in a number of cancer hallmarks. These apparently discrepant results are not without precedent. The novel KRAS mutant E31D, which also harbored a conserved amino acid substitution, was predicted to be benign in all in silico prediction platforms used, but showed highly oncogenic properties in all cancer hallmarks tested [57]. In contrast, C901R was predicted to be oncogenic with significant effect on protein function, and yet was only able to alter a subset of the cancer hallmarks assayed. These findings imply the need to consider the cellular context and possible effect on binding partners in predicting the functional impact of protein mutations. Further, the effect of amino acid substitution on folding and protein assembly as well as the position of the altered residue in the folded configuration all merit further analyses.

The present study also aimed to visualize the structural changes induced by the Q661K and C901R rare mutations. Similar to the E545K hotspot mutation, the Q661K rare mutation is found in the helical domain. E545K disrupts the interaction between the helical domain of PIK3CA and the niSH2 domain of p85 alpha (51), and it is hypothesized that Q661K could have the same effect. Meanwhile, the C901R rare mutation is located in the kinase domain. It is hypothesized that C901R enhances the affinity of PIK3CA to the membrane bilayer because kinase domain mutations like H1047R have been found to act in this manner [61]. The basic arginine residue introduced by C901R could possibly increase the affinity of the protein to the negatively charged phospholipid heads in the membrane.

Analysis of RMSD and OS values revealed that the protein models of the Q661K and C901R rare mutations slightly deviated from the wild type structure. Furthermore, docking simulations with pyDock webserver suggested that the binding affinity of both the rare and canonical mutations with niSH2 domain of p85 alpha decreased, due to their more positive pyDock scores compared with the wild type structure. Analysis of the PIK3CA–p85 alpha interface with PDBePISA suggested that the decrease in binding affinity of the mutations with p85 alpha was due to a loss of a number of salt bridges that were originally present in the wild type-p85 alpha dimer. Altogether, these results suggest that the structural basis for the oncogenic activity of the Q661K and C901R mutations is partly due to a relief from the inhibitory control of the p85 alpha regulatory subunit. Future studies could confirm these results via co-immunoprecipitation and Fluorescence Resonance Energy Transfer (FRET) experiments. In addition, it will be interesting to determine the binding affinity of these mutant proteins with lipid membranes of various compositions through biochemical assays [74]. A complete assessment of these mutations can only be achieved through crystallographic techniques checking for the impact of these mutations on the binding of ligands and additional proteins such as KRAS, downstream effectors, scaffolds, and adaptors. The latter can be complemented by cellular assays confirming their phenotypic effects on cancer hallmarks.

The findings in this study, while instructive, should be confirmed in other cellular backgrounds, in vitro using 3D and co-culture experiments, and in vivo using relevant animal models. Correlating these findings with patient outcomes would also be beneficial in establishing these rare mutations as additional prognostic and predictive biomarkers.

## Figures and Tables

**Table 1:** Primers used for generation of phosphatidylinositol 3-kinase, catalytic subunit alpha (PIK3CA) wild type and mutant constructs.

Primer Name	Sequence
WT-PIK3CA-F	5′-ATGCCTCCACGACCATCATCAGGTGAACTG-3′
WT-PIK3CA-R	5′-TCAGTTCAATGCATGCTGTTTAATTGTGTGGAAG-3′
E545K-F	5′CTCTCTCTGAAATCACT**A**AGCAGGAGAAAGATTTTCTATG-3′
E545K-R	5′-CATAGAAAATCTTTCTCCTGCT**T**AGTGATTTCAGAGAGAG-3′
H1047R-R	5′-GTGACTACTAGTTCAGTTCAATGCATGCTGTTTAATTGTGTGGA AGATCCAATCCATTTTTGTTGTCCAGCCACCATGA**C**GTGCATC-3′
Q661K-F	5′-GAAAGCATTGACTAAT**A**AAAGGATTGGGCACTTTTTCTTTTG-3′
Q661K-R	5′-GAAAAAGTGCCCAATCCTTT**T**ATTAGTCAATGCTTTCTTC-3′
C901R-F	5′-CCTGTTTACACGTTCA**C**GTGCTGGATACTGTGTAGCTACC-3′
C901R-R	5′-GGTAGCTACACAGTATCCAGCAC**G**TGAACGTGTAAACAGG-3′

Note: Bold letters correspond to mutated nucleotide.

**Table 2:** Functional effects of the Q661K and C901R rare mutations as predicted by multiple bioinformatics platforms.

Mutation	Polyphen-2	Mutation Assessor	FATHMM	SIFT
Q661K ^a^	Benign	Neutral	Cancer	Tolerated
C901R ^b^	Probably damaging ^c^	High	Cancer	Affect protein function

^a^ Scores for Q661K: 0.024 (polyphen-2), 0.63 (mutation assessor), −1.63 (FATHMM), 0.46 (SIFT). ^b^ Scores for C901R: 1.00 (polyphen-2), 3.89 (mutation assessor), −1.81 (FATHMM), 0.00 (SIFT). ^c^ Introduced substitution is predicted to be damaging with high confidence [56]. Polyphen-2: polymorphism phenotyping 2; FATHMM: Functional Analysis Through Hidden Markov Models; SIFT: Sorting Intolerant From Tolerant; PIK3CA: phosphatidylinositol 3-kinase, catalytic subunit alpha.

**Table 3:** Analysis of interactions between interface residues of the PIK3CA-niSH2 p85 alpha dimers as predicted by PDBePISA.

	Buried area, Å2	No. of H-Bonds	No. of Salt Bridges	No. of Interfacing Residues in PIK3CA	%	No. of Interfacing Residues in p85 Alpha	%
WT	3756.4	40	31	106	10.70%	106	38.30%
E545K	3673.5	42	27	102	9.70%	105	37.90%
H1047R	4107.8	48	27	119	11.30%	115	41.50%
Q661K	4097.6	46	26	120	11.40%	116	41.90%
C901R	4078.4	44	28	114	10.80%	115	41.50%

## Supplementary Materials

The following are available online at https://www.mdpi.com/2073-4409/9/5/1116/s1, Figure S1: Expression of wild type and mutant PIK3CA gene constructs in HCT116 cells, Figure S2: Western blotting detection of epithelial-to-mesenchymal transition markers.
