# Supplementary material for: Mutation-Associated Phenotypic Heterogeneity in Novel and Canonical PIK3CA Helical and Kinase Domain Mutants
Source: Cells. 2020 Apr 30;9(5):1116. doi: 10.3390/cells9051116 (PMC7290687; doi:10.3390/cells9051116)
Supplement: Supplementary file 1 [file cells-09-01116-s001.zip › Supplementary Figure S1.pdf]

Mutation-associated phenotypic heterogeneity in novel and canonical PIK3CA helical and kinase domain mutants

Arman Ali Ghodsinia [1,†], J-Ann Marie T. Lego [1] and Reynaldo L. Garcia [1,*]

[1] Disease Molecular Biology and Epigenetics Laboratory, National Institute of Molecular Biology and Biotechnology, University of the Philippines Diliman, Quezon City 1101, Philippines

* Correspondence: reygarcia@mbb.upd.edu.ph

† Present address: MRC Oxford Institute for Radiation Oncology, Department of Oncology, Old Road Campus Research Building, Roosevelt Drive, University of Oxford, Oxford, OX3 7DQ, United Kingdom

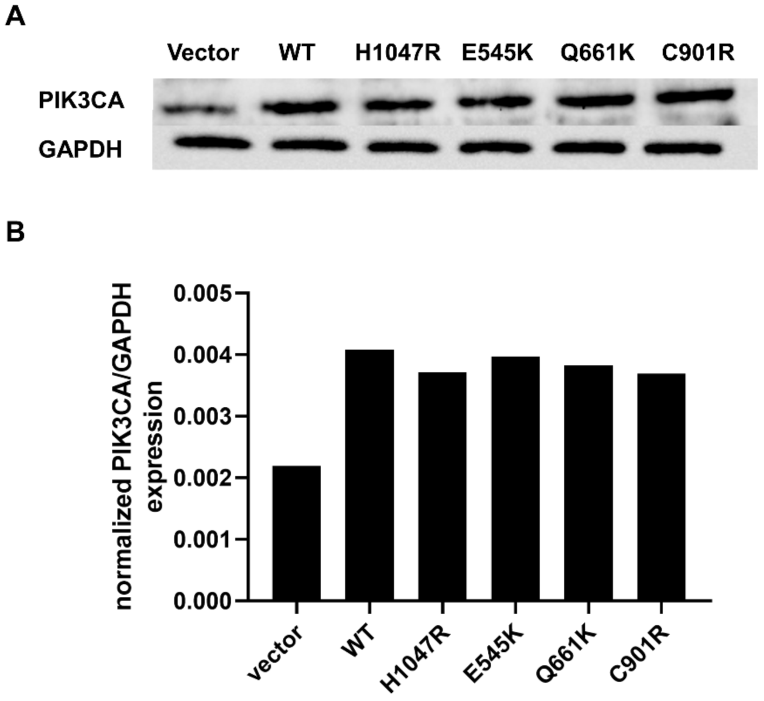

Supplementary Figure S1. Expression of wild type (WT) and mutant PIK3CA gene constructs in HCT116 cells. (A) Western blot detection of protein expression levels of WT and mutant PIK3CA expressed in HCT116 cells. (B) Band intensities were normalized against GAPDH expression
