# Supplementary material for: Mutation-Associated Phenotypic Heterogeneity in Novel and Canonical PIK3CA Helical and Kinase Domain Mutants
Source: Cells. 2020 Apr 30;9(5):1116. doi: 10.3390/cells9051116 (PMC7290687; doi:10.3390/cells9051116)
Supplement: Supplementary file 1 [file cells-09-01116-s001.zip › Supplementary Figure S2.pdf]

Mutation-associated phenotypic heterogeneity in novel and canonical PIK3CA helical and kinase domain mutants

Arman Ali Ghodsinia [1,†], J-Ann Marie T. Lego [1] and Reynaldo L. Garcia [1,*]

[1] Disease Molecular Biology and Epigenetics Laboratory, National Institute of Molecular Biology and Biotechnology, University of the Philippines Diliman, Quezon City 1101, Philippines
* Correspondence: reygarcia@mbb.upd.edu.ph
† Present address: MRC Oxford Institute for Radiation Oncology, Department of Oncology, Old Road Campus Research Building, Roosevelt Drive, University of Oxford, Oxford, OX3 7DQ, United Kingdom

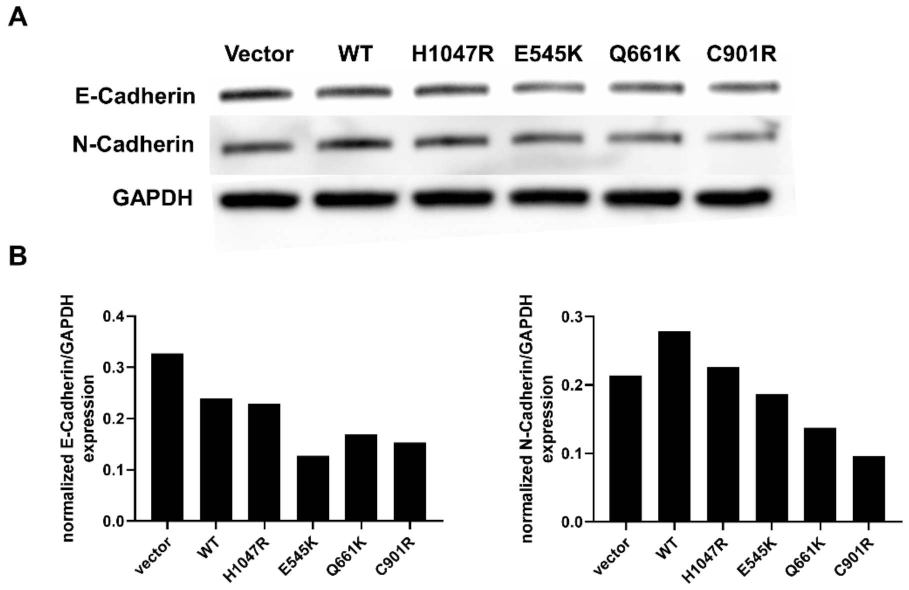

Supplementary Figure S2. Western blotting detection of epithelial-to-mesenchymal transition markers. (A) E- and N-cadherin expression levels in HCT116 cells transfected with wild type and mutant PIK3CA gene constructs. (B) Band intensities of E- and N-cadherin were normalized against GAPDH expression.
